# Challenges in Recruiting University Students for Web-Based Indicated Prevention of Depression and Anxiety: Results From a Randomized Controlled Trial (ICare Prevent)

**DOI:** 10.2196/40892

**Published:** 2022-12-14

**Authors:** Felix Bolinski, Annet Kleiboer, Koen Neijenhuijs, Eirini Karyotaki, Reinout Wiers, Lisa de Koning, Corinna Jacobi, Anna-Carlotta Zarski, Kiona K Weisel, Pim Cuijpers, Heleen Riper

**Affiliations:** 1 Department of Clinical, Neuro, and Developmental Psychology Amsterdam Public Health Research Institute Vrije Universiteit Amsterdam Amsterdam Netherlands; 2 Department of Mental Health and Prevention Trimbos Institute, Netherlands Institute for Mental Health and Addiction Utrecht Netherlands; 3 Department of Research and Business Development HumanTotalCare Utrecht Netherlands; 4 Department of Psychology and Centre for Urban Mental Health University of Amsterdam Amsterdam Netherlands; 5 Department of Clinical Psychology and Psychotherapy Technical University Dresden Dresden Germany; 6 Department of Sport and Health Science Technical University Munich Munich Germany; 7 Department of Clinical Psychology and Psychotherapy Friedrich Alexander University Erlangen-Nuremberg Germany; 8 International Institute for Psychotherapy Babeș-Bolyai University Cluj-Napoca Romania; 9 Department of Psychiatry Amsterdam UMC location VUmc Amsterdam Netherlands

**Keywords:** digital mental health, students, indicated prevention, recruitment, randomized trial, mobile phone

## Abstract

**Background:**

Depression and anxiety are common mental health conditions in college and university student populations. Offering transdiagnostic, web-based prevention programs such as ICare Prevent to those with subclinical complaints has the potential to reduce some barriers to receiving help (eg, availability of services, privacy considerations, and students’ desire for autonomy). However, uptake of these interventions is often low, and accounts of recruitment challenges are needed to complement available effectiveness research in student populations.

**Objective:**

The aims of this study were to describe recruitment challenges together with effective recruitment strategies for ICare Prevent and provide basic information on the intervention’s effectiveness.

**Methods:**

A 3-arm randomized controlled trial was conducted in a student sample with subclinical symptoms of depression and anxiety on the effectiveness of an individually guided (human support and feedback on exercises provided after each session, tailored to each participant) and automatically guided (computer-generated messages provided after each session, geared toward motivation) version of ICare Prevent, a web-based intervention with transdiagnostic components for the indicated prevention of depression and anxiety. The intervention was compared with care as usual. Descriptive statistics were used to outline recruitment challenges and effective web-based and offline strategies as well as students’ use of the intervention. A basic analysis of intervention effects was conducted using a Bayesian linear mixed model, with Bayes factors reported as the effect size.

**Results:**

Direct recruitment through students’ email addresses via the central student administration was the most effective strategy. Data from 35 participants were analyzed (individually guided: n=14, 40%; automatically guided: n=8, 23%; care as usual: n=13, 37%). Use of the intervention was low, with an average of 3 out of 7 sessions (SD 2.9) completed. The analyses did not suggest any intervention effects other than anecdotal evidence (all Bayes factors_10_≤2.7).

**Conclusions:**

This report adds to the existing literature on recruitment challenges specific to the student population. Testing the feasibility of recruitment measures and the greater involvement of the target population in their design, as well as shifting from direct to indirect prevention, can potentially help future studies in the field. In addition, this report demonstrates an alternative basic analytical strategy for underpowered randomized controlled trials.

**Trial Registration:**

International Clinical Trials Registry Platform NTR6562; https://tinyurl.com/4rbexzrk

**International Registered Report Identifier (IRRID):**

RR2-10.1186/s13063-018-2477-y

## Introduction

### Background

Offering transdiagnostic prevention programs for depression and anxiety to college and university students (henceforth denoted as students) is relevant for a number of reasons: these conditions are highly comorbid [1,2] and often emerge during the time individuals enter tertiary education [3-6]. Moreover, symptoms of depression and anxiety are highly correlated with various stressors specifically affecting students, such as considerable academic demands [7,8]; balancing life, studies, and student jobs [9,10]; and, more recently, the global COVID-19 pandemic and the measures implemented to reduce its spread [11,12]. In addition, some studies have reported that financial concerns, in particular student debt, might negatively affect students’ mental health [13,14].

Results from the World Health Organization World Mental Health International College Student initiative [15] suggest that approximately one-third of the almost 14,000 surveyed students had had a mental health disorder in the previous year, with major depressive disorder (MDD; 18.5%) and generalized anxiety disorder (GAD; 16.7%) being the most prevalent conditions [5]. Both disorders cause adverse effects on quality of life [16] and can lead to severe interpersonal impairment. For example, research indicates that more than half of the first-year students who presented with depression or anxiety reported severe disturbances in their social lives and close relationships [17]. In addition, mental health complaints negatively affect students’ academic performance and increase dropout [18,19]. A recent study among Dutch adolescents also highlighted the economic costs of subclinical depression, including health care and societal costs related to school absenteeism [20]. Considering the importance of higher education for economic growth [21], preventing the onset of mental health conditions in students matters from an individual, societal, and economic perspective.

In particular, indicated prevention approaches [22] that focus on individuals with subclinical complaints could be beneficial. These have the potential to prevent the onset of, for example, MDD [23] and—in contrast to universal approaches—reduce expenditure on costly treatment by allocating scarce health care resources to those students who are in immediate need [24]. Meta-analyses of community samples suggest that preventive interventions can reduce the incidence rate of depression by approximately 19% [25] and of anxiety by approximately 43% [26].

Despite the need for and availability of such interventions [27,28], uptake of preventive programs is particularly low compared with treatment for psychological disorders [29,30], likely as perceived symptoms are not severe enough yet to motivate help seeking. Students in particular often do not seek professional help—merely approximately a quarter of World Health Organization International College Student Initiative respondents indicated that they would definitely seek treatment for mental health complaints; of those who would not seek help, approximately half indicated that they would rather deal with the problem on their own or preferred to consult with friends or relatives [31]. Fear of stigmatization is another reason for low help-seeking behavior [32,33]. In addition, on-campus mental health services often lack the capacity to meet the needs of those students who seek help [34]. Moreover, often these services focus exclusively on study-related issues (eg, test anxiety and procrastination). As a result, studies suggest that only approximately one-third to one-sixth of students receive adequate help for their complaints [35,36].

Digital interventions provided via the internet on computers or via smartphone apps have been proposed as a way to overcome barriers to the availability of counseling services and help seeking by offering the privacy and autonomy desired by students [37]. A recent meta-analysis on the effectiveness of such interventions for mental health complaints included 48 randomized controlled trials (RCTs) on individually guided (ie, tailored human support) and unguided or automatically guided (ie, computer-generated standardized feedback) digital interventions, most of them based on cognitive behavioral therapy [28]. Small but significant differences favoring the interventions were found only when these were compared with passive controls (eg, depression: *g*=0.18; anxiety: *g*=0.27), with individual guidance having no significant effect on these results. However, slightly larger effects on depression outcomes (*g*=0.29) were found in studies that targeted subclinical complaints. Importantly, the prediction intervals in this meta-analysis suggest that future trials will likely include nil effects [28]. In addition, transdiagnostic components that target both conditions may be beneficial, particularly for depression outcomes (*g*=0.22) [38]. Although this indicates some potential for indicated transdiagnostic prevention efforts, the general focus on reduction of symptoms rather than prevention of (future) mental health conditions implies a need for further research [39]. However, an RCT has shown that a digital intervention can also prevent the onset of MDD in the general population [23].

### Objectives

On the basis of these considerations, ICare Prevent—a transdiagnostic individually tailored digital intervention for the indicated prevention of depression and anxiety—was developed. We planned to conduct an RCT on the effectiveness of the intervention among students in the Netherlands. However, despite 2 years of extensive countrywide recruitment efforts, this trial was concluded without reaching the targeted sample size (N=252). Therefore, the aims of this study are 3-fold, namely to (1) describe the recruitment process for the RCT, (2) describe participants’ use of the intervention, and (3) conduct a basic analysis of intervention effects on depressive and anxiety symptoms.

## Methods

### General Study Design and Inclusion and Exclusion Criteria

This trial was registered in the International Clinical Trials Registry Platform (NTR6562), and a detailed protocol for the planned trial has been published [40]. In summary, the design entailed a 3-arm parallel superiority trial comparing an individually guided and an automatically guided version of the intervention with care as usual (CAU). The aim was to include 252 students (84 per condition; expected effect size: *d*=0.35; for the power calculation, see the protocol by Bolinski et al [40]). Recruitment started in June 2017 and was concluded in July 2019.

Dutch- and English-speaking students aged ≥16 years with subclinical symptoms of depression or anxiety, defined as a score of ≥16 on the Center for Epidemiological Studies Depression Scale [41] or ≥5 on the 7-item Generalized Anxiety Disorder Scale (GAD-7) [42], respectively, could participate if they provided written informed consent. They were excluded if they were in remission of a major depressive episode; currently meeting diagnostic criteria for a mood or anxiety, lifetime bipolar, or psychotic disorder; or presenting moderate to high suicide risk (all assessed by phone using the Mini International Neuropsychiatric Interview [MINI]) [43]. In addition, they were excluded if they reported currently being on a waitlist for or having received psychotherapy in the previous half year as well as participating in similar intervention studies at the time of the screening assessment.

Randomization was performed at the individual level at a 1:1:1 allocation ratio following baseline assessment. It was stratified by the type of subclinical symptoms (ie, depression, anxiety, or both according to the MINI). Subsequent assessment points were the midway point (5 weeks after randomization), posttest assessment (8 weeks after randomization), and the 6- and 12-month follow-ups.

### Procedure

**Figure 1:**
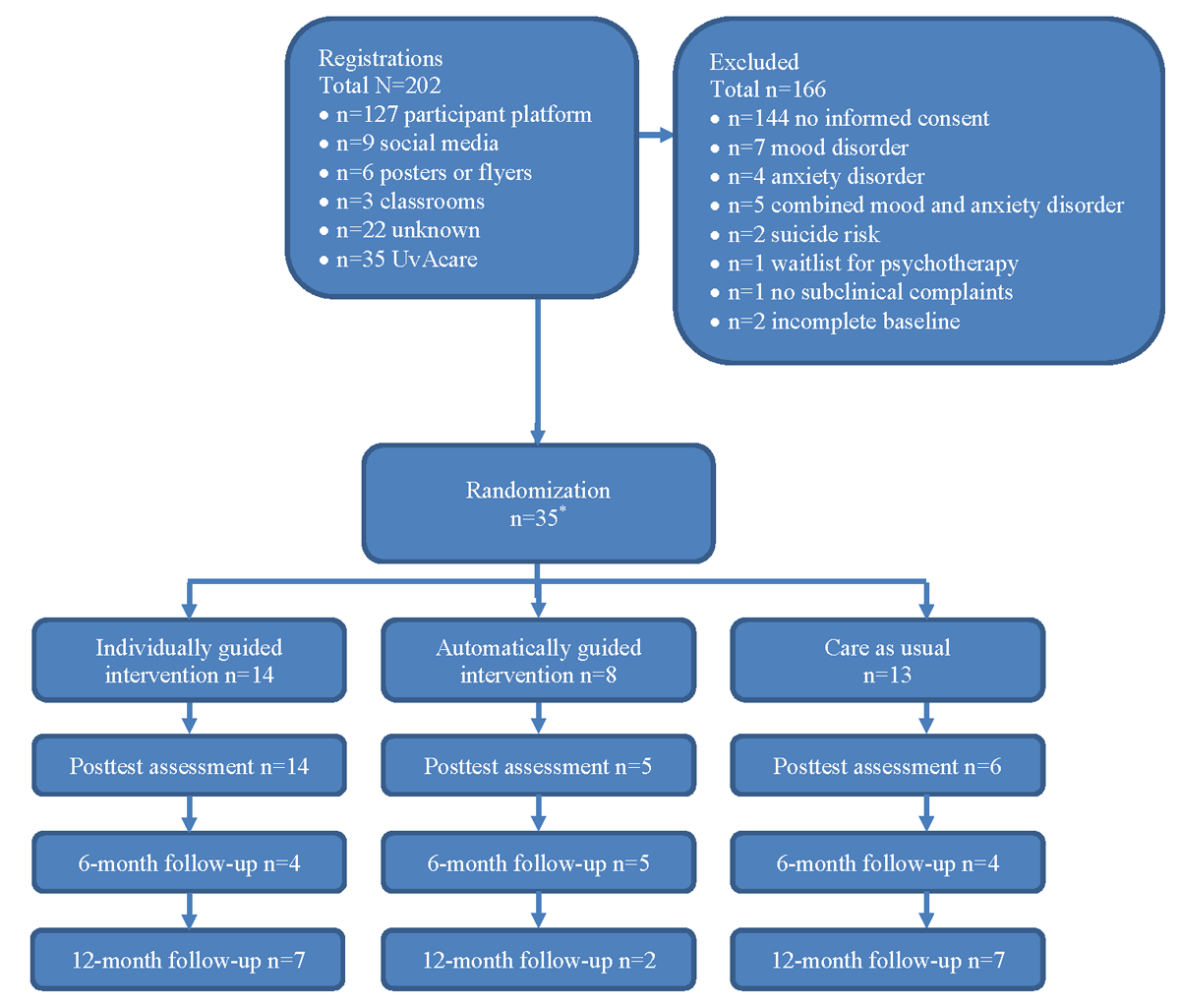
Study flowchart (*=1 participant excluded because of dropout after randomization).

Participants first received information on the study. Following registration and provision of written informed consent, self-reported inclusion and exclusion criteria were assessed through web-based questionnaires, followed by the assessment of diagnostic exclusion criteria by phone. Participants were then randomized according to incoming informed consent forms by an independent researcher using a dedicated randomization platform. Owing to the nature of the study, blinding of participants and of the coaches who provided guidance in the individually guided intervention arm was not possible. However, assessors performing the MINI interviews at the posttest and follow-up assessments were blind to the participants’ group allocation (see the flowchart in Figure 1).

### Recruitment Methods

A broad array of recruitment measures was used, such as social media campaigns, printed advertisements, a paid participant platform, and other project collaborations. Examples of some of the recruitment materials are provided in Multimedia Appendices 1 and 2.

#### Social Media

Paid social media campaigns on Facebook and Instagram were targeted to higher-education students in the Netherlands. These advertisements contained images that reflected the study aim, a brief outline of the web-based intervention, and a call to visit the study website and register for participation. In addition, information on the study and the call to participate were posted on Facebook groups relevant to students.

#### Print and News Media

Flyers, posters, and stickers were distributed at universities countrywide. Similar to social media posts, they contained brief information on the study and the intervention as well as contact details (study website and email address). This material was also published in 2 of the largest student newspapers.

#### Targeted Email Distribution Through Project Collaboration (UvAcare)

In March 2019, recruitment was extended to a student mental health project conducted at another university (UvAcare) [44]. Therein, all students were screened for mental health complaints, including those with more severe presentations of such problems. Screening generally took place at the beginning of a semester. A data-sharing agreement was set up, and data on demographic variables and primary mental health outcomes of a subsample of participating (PhD) students fulfilling the aforementioned inclusion criteria were provided. This collaboration offered the possibility of using students’ email addresses—sent through the central administration—for providing information on the study and the intervention and a digital rather than written informed consent form. In this project collaboration, the use of email addresses remained the primary recruitment channel. A full description of this project is provided in the associated study protocol [44].

#### Participant Platform

A paid platform for participation in clinical trials was used [45]. The platform maintains a directory of users who are interested in participating in clinical trials. Information on the study was uploaded to the platform, and interested users could sign up to receive the information letter and informed consent form. A brief questionnaire on whether a user was currently registered at a Dutch higher education institution and not currently receiving psychological treatment was implemented as an initial screener.

#### Other

Key individuals at the university (eg, student bodies, teachers, and counselors) were approached to create support for the study. It was then pitched in classrooms and web-based education portals. Awareness of the project was also created by participating in the largest running event in the Netherlands and wearing a shirt printed with the study website [46]. Local and international conferences were used to generate a network of contacts that could help in the dissemination of project information and, thus, recruitment.

### Intervention

ICare Prevent is a web-based and mobile-supported intervention for the indicated prevention of depression and anxiety. It uses both transdiagnostic and individually tailored elements and was originally developed for the German-speaking general population following existing evidence- and cognitive behavioral therapy–based web-based modules from different digital interventions [23,47]. The intervention comprises a sequential (ie, a session has to be completed to unlock the next) 7-session web-based program with 1 booster session. Participants were advised to complete between 1 and 2 sessions per week, with each requiring approximately 45 to 60 minutes to be completed. In addition, 8 elective modules (on sleep, perfectionism, gratitude, self-esteem, alcohol use, relaxation, acceptance, and rumination) and 5 diaries (positive activities, negative thoughts, sleep, challenging situations, and alcohol use) targeted factors common to both mood and anxiety problems. All the sessions and elective modules included individual exercises that needed to be completed. In line with its transdiagnostic, individually tailored approach, users received information targeting both conditions (sessions 1-4) before they could prioritize the techniques that focused on their most prominent complaints (sessions 5 and 6).

In the individually guided arm, clinical psychology students motivated participants to continue with the sessions and provided structured and personalized feedback on the homework exercises. Those allocated to the automatically guided intervention received standard and computerized feedback after completing each session, which was geared toward motivating them to continue to the next session. Technical and usability questions could be asked by participants in either group.

As part of the study, the intervention was translated and adapted to the student context in the Netherlands. To do this, the first author worked together with a graphic designer and text editor experienced in web editing. Finally, the intervention was tested on spelling and functionality by a number of students who were employed as research assistants. Adaptations included changing the testimonials to be more representative of a diverse student population, changing the focus of exercises to problems more applicable to students, and considerably shortening the amount of text in the modules while keeping the structure and main content of the original intervention intact (see Multimedia Appendix 3 and the protocol by Bolinski et al [40] for a description of the intervention content per session and its transdiagnostic components). Students in the CAU condition were informed that they could seek help from their general practitioner (GP) or student psychologist for any mental health complaints. However, students in all trial arms were free to access CAU during the study.

### Primary Outcome Variables

In total, 2 primary outcome variables were assessed by interviewers via telephone to measure changes in disorder-specific symptoms from baseline to posttest and follow-up assessments [40]. For depression, the clinician-rated version of the Quick Inventory of Depressive Symptomatology (QIDS-CR) [48] was used. This 16-item instrument assesses 9 symptom criteria for depression (sad mood, concentration difficulties, self-criticism, suicidal thoughts, general interest, fatigue, sleep, appetite, and psychomotor retardation) based on the Diagnostic and Statistical Manual of Mental Disorders, Fourth Edition [49], with total scores ranging from 0 to 27. Higher scores indicate greater psychopathology. The QIDS-CR has well-established psychometric properties and is sensitive to the effects of treatment [50,51]. It has been shown to generate reliable scores when administered over the phone compared with in-person or paper-and-pencil versions [52]. Anxiety was assessed using the Structured Interview Guide for the Hamilton Anxiety Rating Scale (SIGH-A) [53,54]. A total of 14 items cover anxiety symptoms, resulting in a total score ranging from 0 to 56, with higher scores again indicating greater psychopathology. The SIGH-A has well-established psychometric properties and has provided sensitive diagnoses compared with, for example, in-person ratings [53,55,56]. More information on the instruments has been provided in the study protocol [40].

### Other Variables

#### Overview

The original study protocol [40] lists a number of secondary variables. In this report, only data collected through the project collaboration (UvAcare) could be analyzed. Therefore, only a subset of secondary variables that had also been collected in the UvAcare project is reported here.

#### Psychopathology

In addition to during screening, the MINI was also completed over the phone during follow-up assessments. The interview has reasonably good psychometric properties [43,57,58]. In addition, 2 self-report questionnaires were administered at all assessment points after screening. The 9-item Patient Health Questionnaire (PHQ-9) [59] measures the presence of depressive symptoms during the past 2 weeks on a 4-point Likert scale (0=not at all to 3=nearly every day). The PHQ-9 is a reliable and valid screening instrument with established cutoff scores of 5, 10, and 20 referring to mild, moderate, and severe depression, respectively [59]. The GAD-7 is scored identically and measures anxiety symptoms during the past 2 weeks, with total values of 5, 10, and 15 indicating mild, moderate, and severe manifestations of generalized anxiety, respectively [42]. The questionnaire has good psychometric properties [60].

#### Health Care Use

At posttest assessment and during follow-up assessments, CAU use was monitored by asking participants if they had been in contact with any health care professionals (eg, GP, student counseling services, or psychologist) in the previous 2 months. If so, they were asked to indicate how often they had had contact with the professional. However, the reasons for their visits could not be established.

#### Intervention Adherence

Log data were retrieved from the intervention platform. Given the sequential nature of the intervention, the last completed main module was taken as an indication of intervention progress. Furthermore, the number of diary entries and completed elective modules was retrieved.

### Reporting and Statistical Analysis

Recruitment data were used to target the first aim of this study. Where this was traceable (eg, through web statistics or information from the participants), the number of potential participants reached and the number of registrations were reported per recruitment method. Subsequent progress, such as returned informed consent forms and final inclusions, was described. Study dropout was reported per condition and assessment point after baseline by calculating the percentage of included participants who did not complete the interviews and self-report questionnaires. Descriptive statistics for all assessed variables were calculated. Intervention and health care use were reported to target the second aim of this report. This comprises the average number of completed sessions, the percentage of elective modules chosen over the entirety of the sessions (as elective modules could be completed multiple times across sessions), and diary entries, as well as the number of students who visited a health care professional and their contact frequency. For the individually guided intervention condition, the median and average number of messages sent between coaches and participants was calculated.

Finally, to tackle the third aim, we carried out a basic analysis of intervention effectiveness on the intention-to-treat sample under a Bayesian framework. If not otherwise specified, all statistical analyses were run in R (R Foundation for Statistical Computing) and RStudio (version 4.1.1; RStudio, Inc [61]; references for auxiliary packages used are provided at the end of this report). A Bayesian linear mixed model using BRMS (version 2.17.0; Bürkner et al [62]) and RStan (version 2.21.5; Guo et al [63]), including both random and fixed effects (chains=4, iterations=4000, and burn-in phase=1000), was conducted separately for the 2 primary outcomes, the QIDS-CR and SIGH-A, whereby all 4 assessment points were nested within individuals. Gender was entered as a covariate. The analyses were repeated for the self-reported measures of depression (PHQ-9) and anxiety (GAD-7), which contained an additional assessment point (midway). The Bayesian approach—in addition to other advantages (see the articles by Wagenmakers et al [64] and by Verhagen and Wagenmakers [65] for a summary)—allowed us to still provide information on the distribution of the small data set without the power restrictions inherent in the frequentist framework. However, to increase the explanatory value of the data, participants from both intervention groups (ie, individually guided and automatically guided) were pooled to constitute 1 intervention condition, which was then compared with CAU in the analyses through an interaction term with assessment point (for a discussion on the results of a sensitivity analysis including all 3 groups separately, see Multimedia Appendix 4). At each assessment point, the hypothesis that the interaction effect (ie, an intervention effect) was different from zero was tested using contrasts comparing each assessment point with the baseline. Moreover, no prior information was included in the analysis, resulting in flat prior distributions [66]. The probability of the collected data emerging under the null model (ie, meaning that the data were a collection of random noise) was compared with the alternative model (ie, condition [intervention vs CAU] had an effect on outcome). The resulting conditional probability was the Bayes factor (BF), which was reported as a measure of the size of the effect for each postbaseline assessment point. Both notations of the BF are reported: BF_10_, indicating support for the alternative over the null hypothesis, and BF_01_, indicating support for the null over the alternative hypothesis [67]. When considering the strength of the support, a BF_10_ of approximately 1 indicates no support, and a BF_10_ of >10 indicates strong support [68]. High-density intervals were calculated to accompany credibility intervals (CIs) as a measure of the uncertainty of the estimated parameters. Finally, the results of the MINI were used to report the number of individuals who met the diagnostic criteria for a mood or anxiety disorder at the 2 follow-up assessments.

### Ethics Approval

The ICare Prevent trial was registered in the International Clinical Trial Registry Platform (ICare Prevent NTR6562) and approved by the medical ethics committees of the Amsterdam University Medical Center (NL6075.029.17 and A2018.166). All participants had to provide informed consent upon registration for the study. A data-sharing agreement between the 2 universities involved in this report was set up. This report has been compiled in accordance with the CONSORT-EHEALTH (Consolidated Standards of Reporting Trials of Electronic and Mobile Health Applications and Online Telehealth) guidelines [69].

## Results

### Recruitment and Screening

The reach of the recruitment activities could only be determined with certainty for targeted social media advertisements (ie, Facebook and Instagram), defined as the number of individuals who were presented with the advertisement on their computer or smartphone. Reach for other channels such as printed advertisements and sports events was estimated based on publicly available information (eg, number of students and number of attendees). Table 1 provides an overview of the reach and number of registrations per recruitment method, with most registrations stemming from the participant platform (127/202, 62.9%).

Of the 167 students who registered for participation outside of the project collaboration (UvAcare), only 23 (13.8%) returned the signed informed consent form and were assessed for eligibility. Of these 23 individuals, 18 (78%) were excluded at screening based on elevated psychopathology on the MINI (mood disorder: n=7, 39%; anxiety: n=4, 22%; combined mood and anxiety disorder: n=5, 28%; suicide risk: n=2, 11%), 1 (6%) was excluded for being on a waitlist for psychotherapy, and another (6%) was excluded because complaints were too light. This left 13% (3/23) of inclusions, one of which completed the baseline assessment and was randomized. However, this participant dropped out of the study before the midintervention assessment and, thus, only provided baseline data (not reported in this manuscript). Finally, data from 35 students were provided through the collaboration with the UvAcare student mental health project. These students were recruited through their email addresses and provided digital informed consent, making this the most successful approach to recruitment.

**Table 1:** Reach of potential participants and subsequent registrations for participation per recruitment method (N=202).

Recruitment method	Reach, N	Registrations, n (%)
Social media (Instagram and Facebook)	122,044^a^	9 (4.5)
Printed advertisements (flyers, posters, and newspapers)	115,000^b^	6 (3)
Classrooms and web-based education platforms	Unknown	3 (1.5)
Participant platform	Unknown	127 (62.9)
Conference presentations	Unknown	Unknown
Running event	11,000^c^	Unknown
Targeted emails (UvAcare)	Unknown	35 (17.3)
Unknown or could not be determined	Unknown	22 (10.9)

^a^Actual number based on data extracted from the Facebook advertisement platform.

^b^Estimate based on official numbers of registered students at Utrecht University, Leiden University, University of Groningen, and University of Amsterdam (2018).

^c^Estimate based on the number of participants according to the official website.

### Participants and Study Dropout

The 35 participants (individually guided intervention: n=14, 40%; automatically guided intervention: n=8, 23%; CAU: n=13, 37%; descriptives in Table 2) had a mean age of 25.86 (SD 4.75) years, with a slight majority of female students (19/35, 54%). A total of 71% (25/35) were undergraduate students, and 29% (10/35) were PhD students.

Study dropout was 57% (20/35; individually guided condition: 8/14, 57%; automatically guided condition: 4/8, 50%; CAU: 8/13, 62%) at the midway assessment, 29% (10/35; individually guided condition: 0%; automatically guided condition: 3/8, 38%; CAU: 7/13, 54%) at the posttest assessment, and 43% (15/35) at both follow-up assessments (6 months: 5/14, 36% in the individually guided condition, 3/8, 38% in the automatically guided condition, and 7/13, 54% in CAU; 12 months: 5/14, 36% in the individually guided condition, 4/8, 50% in the automatically guided condition, and 6/13, 46% in CAU). It is noteworthy that the relatively higher dropout rate at the midassessment was likely related to the fact that it only consisted of a self-report questionnaire and no telephone interview.

**Table 2:** Descriptive statistics of clinical variables per condition and assessment point.

Time point		QIDS-CR^a^, mean (SD)	Participants, n (%)	PHQ-9^b^, mean (SD)	Participants, n (%)	SIGH-A^c^, mean (SD)	Participants, n (%)	GAD-7^d^, mean (SD)	Participants, n (%)
**Total sample (N=35)^e^**									
	Baseline	5.51 (2.84)	35 (100)	7.54 (3.79)	35 (100)	5.77 (4.51)	35 (100)	6.23 (3.72)	35 (100)
	Midway^f^	—^g^	—	5.13 (2.5)	15 (43)	—	—	4.47 (3.7)	15 (43)
	Posttest assessment^h^	4.92 (2.77)	25 (71)	6.0 (5.26)	7 (20)	5.32 (4.95)	25 (71)	4.43 (4.24)	7 (20)
	6-month follow-up	4.92 (2.53)	13 (37)	5.58 (4.56)	19 (54)	3.69 (4.29)	13 (37)	4.63 (3.72)	19 (54)
	12-month follow-up	5.13 (3.59)	16 (46)	5.25 (3.64)	20 (57)	1.06 (1.44)	16 (46)	4.2 (3.82)	20 (57)
**Individually guided condition (n=14)^i^**									
	Baseline	4.79 (2.61)	14 (100)	6.14 (2.03)	14 (100)	4.71 (3.36)	14 (100)	4.93 (3.08)	14 (100)
	Midway	—	—	4.0 (2.9)	6 (43)	—	—	3.17 (2.71)	6 (43)
	Posttest assessment	4.86 (3.23)	14 (100)	3.0 (1.83)	4 (29)	5.57 (5.92)	14 (100)	2.0 (1.83)	4 (29)
	6-month follow-up	5.0 (3.37)	4 (29)	4.22 (2.64)	9 (64)	1.75 (2.06)	4 (29)	3.22 (3.11)	9 (64)
	12-month follow-up	4.14 (2.73)	7 (50)	4.78 (3.56)	9 (64)	1.0 (1.53)	7 (50)	4.11 (3.79)	9 (64)
**Automatically guided condition (n=8)^j^**									
	Baseline	5.88 (2.64)	8 (100)	8.0 (3.82)	8 (100)	6.0 (5.61)	8 (100)	7.5 (4.93)	8 (100)
	Midway	—	—	6.25 (3.2)	4 (50)	—	—	7.0 (5.89)	4 (50)
	Posttest assessment	4.2 (2.05)	5 (62)	N/A^k^	0 (0)	5.0 (4.36)	5 (62)	N/A	0 (0)
	6-month follow-up	3.8 (1.92)	5 (62)	2.75 (3.59)	4 (50)	2.8 (4.38)	5 (62)	3.5 (5.2)	4 (50)
	12-month follow-up	2.0 (0.0)	2 (25)	2.25 (1.71)	4 (50)	0.0 (0.0)	2 (25)	2.25 (4.5)	4 (50)
**CAU^l^ condition (n=13)^m^**									
	Baseline	6.1 (3.23)	13 (100)	8.77 (4.87)	13 (100)	6.77 (4.97)	13 (100)	6.85 (3.36)	13 (100)
	Midway	—	—	5.6 (0.55)	5 (38)	—	—	4.0 (1.87)	5 (38)
	Posttest assessment	5.68 (2.25)	6 (46)	10.0 (6.0)	3 (23)	5.0 (3.29)	6 (46)	7.67 (4.62)	3 (23)
	6-month follow-up	6.25 (2.22)	4 (31)	9.5 (5.28)	6 (46)	6.75 (4.99)	4 (31)	7.5 (1.87)	6 (46)
	12-month follow-up	7.0 (4.04)	7 (54)	7.57 (3.31)	7 (54)	1.43 (1.51)	7 (54)	5.43 (3.55)	7 (54)

^a^QIDS-CR: Quick Inventory of Depressive Symptomatology-Clinician Rated [48].

^b^PHQ-9: Patient Health Questionnaire [59].

^c^SIGH-A: Structured Interview Guide for the Hamilton Anxiety Rating Scale [53,54].

^d^GAD-7: Generalized Anxiety Disorder Scale [42].

^e^Mean age 25.86 (SD 4.75) years; 54% (19/35) women; 46% (16/35) men.

^f^Midway: 5 weeks after randomization; only self-report assessed.

^g^Not available.

^h^Posttest assessment: 8 weeks after randomization.

^i^Mean age 27.86 (SD 6.67) years; 64% (9/14) women; 36% (5/14) men.

^j^Mean age 25.5 (SD 1.41) years; 50% (4/8) women; 50% (4/8) men.

^k^N/A: not applicable.

^l^CAU: care as usual.

^m^Mean age 23.92 (SD 2.4) years; 46% (6/13) women; 54% (7/13) men.

### Intervention and Health Care Use

The 63% (22/35) of participants in the intervention conditions completed an average of 3 sessions (SD 2.9; individually guided condition: mean 4, SD 2.9; automatically guided condition: mean 2, SD 2.4). A total of 7 participants (individually guided condition: n=3, 43%; automatically guided condition: n=4, 57%) started but did not complete the first session, and only 7% (1/14) of the participants in the individually guided condition completed the booster module. In most sessions that offered the selection of an elective module (ie, sessions 2 to 7; 19/49, 39%), no elective module was chosen. Across all these sessions, the most frequently selected module dealt with improving self-esteem (9/49, 18%), followed by gratitude (6/49, 12%), improving sleep, and dealing with perfectionism (5/49, 10% each). The elective module teaching acceptance techniques was chosen in 6% (3/49) of all completed sessions. The relaxation, rumination, and reducing alcohol consumption modules were chosen the least frequently, namely, in 2% (1/49) of all completed sessions each. Only 14% (2/14) of the participants in the individually guided condition used the diary function, with a total of 8 entries in the activity diary. On average, the coaches and participants in this condition exchanged 5 (SD 4.79; median 3) messages.

At posttest assessment, 4 students (individually guided condition: n=2, 50%; CAU: n=2, 50%) indicated that they had consulted one or more health care professionals in the previous 2 months (GP: n=3, 75%; student psychologist or psychotherapist: n=2, 50%; study advisor or counselor: n=2, 50%; other: n=1, 25%). Frequencies ranged from 1 to 3 visits. This increased to 11 students (individually guided condition: n=4, 36%; automatically guided condition: n=3, 27%; CAU: n=4, 36%) at the 6-month follow-up (GP: n=4, 36%; study advisor or counselor: n=4, 36%; student psychologist or psychotherapist: n=4, 36%; self-help group: n=1, 9%; other: n=4, 36%), with frequencies ranging from single to weekly visits (self-help group). At the 12-month follow-up, 10 students (individually guided condition: n=4, 40%; automatically guided condition: n=2, 20%; CAU: n=4, 40%) had visited one or more health care professionals (study advisor or counselor: n=1, 10%; student psychologist or psychotherapist: n=5, 50%; other: n=3, 30%). The visits ranged from 1 to 7 times.

### Symptom Change and Diagnoses

The results on symptom change (Table 3) suggest moderate to strong support for the null hypothesis (ie, the absence of an intervention effect) on almost all mental health outcomes at all assessment points (BF_10_ range=0.03-2.7; BF_01_ range=0.37-35.36) [68]. Values of >1 for BF_10_, which indicates support for the hypothesis that an intervention effect is present, were found exclusively in measures of anxiety. Specifically, for the GAD-7, the interaction between condition and time (β=−0.39, 95% CI −3.78 to 2.91; BF_01_=0.7) was 1.43 times more likely to emerge under the alternative hypothesis than under the null hypothesis at the midway assessment compared with the baseline. For the SIGH-A, this interaction was found to be 1.64 times more likely at posttest assessment versus baseline (β=−0.82, 95% CI −6.18 to 4.52; BF_01_=0.61) and 2.7 times more likely at the 12-month follow-up versus baseline (β=−1.36, 95% CI −5.86 to 3.13; BF_01_=0.37) under the alternative hypothesis.

**Figure 2:**
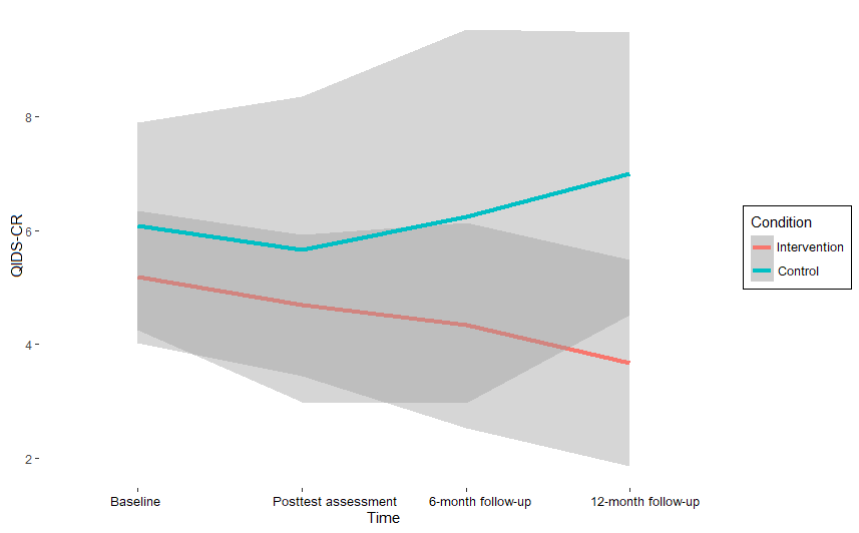
Depression scores (Quick Inventory of Depressive Symptomatology-Clinician Rated [QIDS-CR]) per assessment point and pooled condition with 95% credibility intervals.

**Figure 3:**
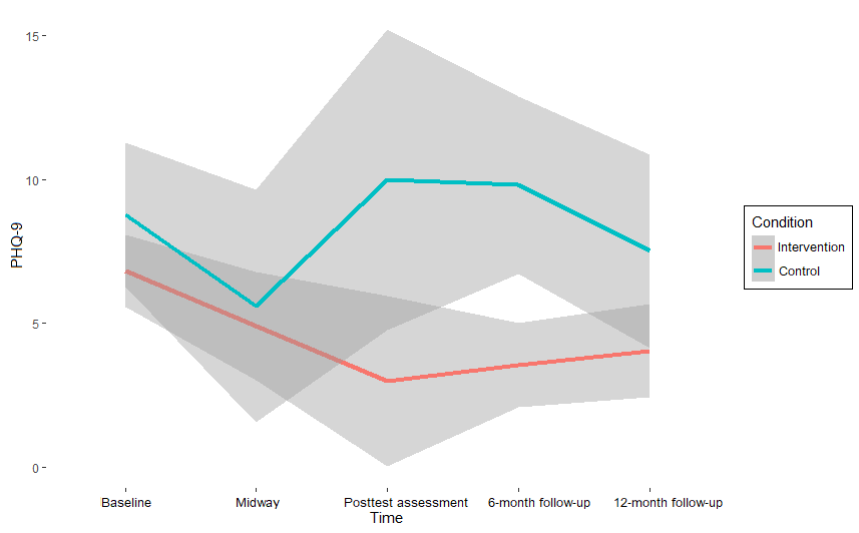
Self-reported depression scores (Patient Health Questionnaire [PHQ-9]) per assessment point and pooled condition with 95% credibility intervals.

**Figure 4:**
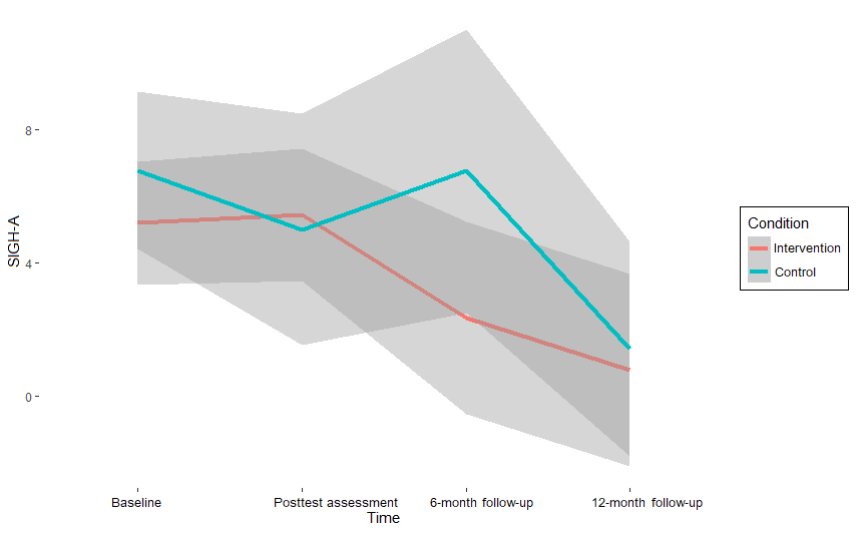
Anxiety scores (Structured Interview Guide for the Hamilton Anxiety Rating Scale [SIGH-A]) per assessment point and pooled condition with 95% credibility intervals.

**Figure 5:**
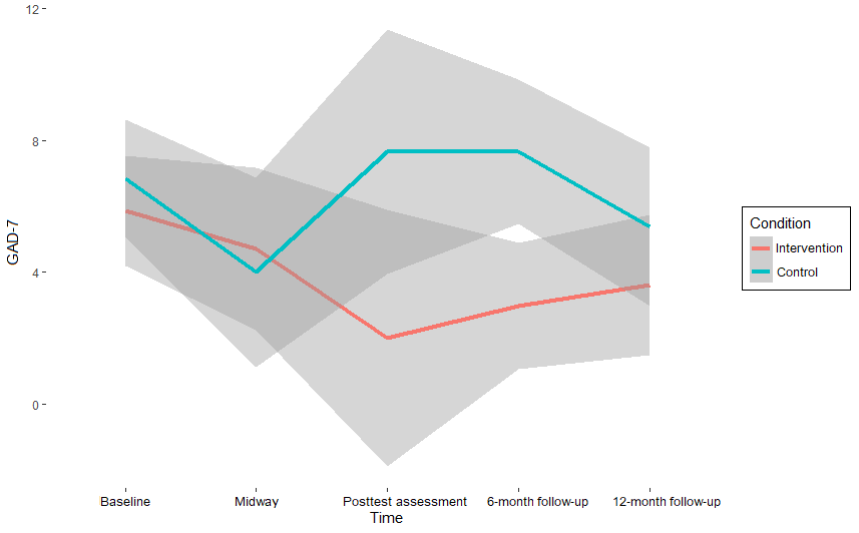
Self-reported anxiety scores (Generalized Anxiety Disorder Scale [GAD-7]) per assessment point and pooled condition with 95% credibility intervals.

Although these effects indicate a superior effect of CAU over the intervention condition, as CAU shows stronger reductions in symptoms compared with baseline at these assessment points, the strength of the support is only anecdotal [68] and is associated with significant uncertainty, as indicated by both the CIs and high-density intervals, reflecting the small sample size (see also Figures 2-5). Thus, there is no reliable evidence of an intervention effect (Table 3).

At the 6-month follow-up assessment, 13 students completed the telephone interviews, of whom 1 (8%) in the individually guided intervention condition presented with a GAD according to the MINI. In total, 16 students completed the 12-month follow-up interviews, of whom 2 (12%) in the CAU condition met diagnostic criteria: 1 (50%) for dysthymia and another (50%) for GAD. Another 14% (2/14) of the students in the individually guided condition presented with mixed anxiety and depression.

**Table 3:** Estimates and Bayes factors (BFs) for the interaction with condition per assessment point and instrument.

Time point			Estimate, β		95% CI^a^		95% HDI^b^		BF_10_^c^		BF_01_^d^
**QIDS-CR^e^**											
	Baseline vs posttest assessment	1.25		−1.63 to 4.09		−1.62 to 4.10		0.23		4.36	
	Baseline vs 6-month follow-up	2.29		−1.19 to 5.79		−1.00 to 5.96		0.10		10.18	
	Baseline vs 12-month follow-up	2.21		−0.65 to 5.04		−0.58 to 5.08		0.06		15.67	
**PHQ-9^f^**											
	Baseline vs midway	.34		−4.47 to 5.13		−4.65 to 4.93		0.78		1.28	
	Baseline vs posttest assessment	3.36		−5.12 to 11.6		−5.42 to 11.27		0.22		4.54	
	Baseline vs 6-month follow-up	4.10		−1.24 to 9.44		−1.43 to 9.21		0.06		15.78	
	Baseline vs 12-month follow-up	1.70		−2.21 to 5.60		−2.10 to 5.70		0.23		4.30	
**SIGH-A^g^**											
	Baseline vs posttest assessment	−.82		−6.18 to 4.52		−6.13 to 4.54		1.64		0.61	
	Baseline vs 6-month follow-up	3.94		−2.15 to 10.18		−2.39 to 9.90		0.10		9.88	
	Baseline vs 12-month follow-up	−1.36		−5.86 to 3.13		−5.92 to 3.07		2.70		0.37	
**GAD-7^h^**											
	Baseline vs midway	−.39		−3.78 to 2.91		−3.78 to 2.91		1.43		0.70	
	Baseline vs posttest assessment	3.07		−3.53 to 9.64		−3.79 to 9.28		0.16		6.43	
	Baseline vs 6-month follow-up	3.23		−0.09 to 6.53		0.09 to 6.69		0.03		35.36	
	Baseline vs 12-month follow-up	.68		−3.07 to 4.60		−3.09 to 4.57		0.56		1.79	

^a^CI: credibility interval.

^b^HDI: high-density interval.

^c^BF_10_: BF indicating probability of alternative over null hypothesis.

^d^BF_01_: BF indicating probability of null over alternative hypothesis.

^e^QIDS-CR: Quick Inventory of Depressive Symptomatology-Clinician Rated [48].

^f^PHQ-9: Patient Health Questionnaire [59].

^g^SIGH-A: Structured Interview Guide for the Hamilton Anxiety Rating Scale.

^h^GAD-7: Generalized Anxiety Disorder Scale [42].

## Discussion

Despite the availability of effective low-threshold mental health interventions, such as those provided via the internet [28], students seem to be difficult to recruit for interventions on this topic. Therefore, the aims of this study were to (1) describe the recruitment process for an RCT of a web-based intervention for the prevention of depression and anxiety in students, (2) describe how participants used the intervention, and (3) conduct a basic analysis of intervention effects.

### Recruitment Process

We used an extensive and multifaceted recruitment strategy, ranging from traditional print and social media advertisements to more unconventional means such as participation in a sports event. Ultimately, direct contact through students’ email addresses was the most effective recruitment channel, which was a unique opportunity in the UvAcare project [44]. A larger number of students could be reached efficiently at the same time, although this method focused largely on screening students at the beginning of a semester, when exam anxiety and other stressors might not have set in yet. It is possible that more students would have fulfilled the inclusion criteria toward the end of the semester. However, this strategy potentially reduced the feeling of being individually targeted for mental health complaints. Although, to the best of our knowledge, this has not been tested yet, it is conceivable that—compared with a universal recruitment strategy—an email from the central student administration may have led to more compliant behavior in completing the screener and providing informed consent for the subsequent RCT. Moreover, the use of digital informed consent reduced the barrier to participation in the UvAcare project. Printing and posting a form, as required in the initial trial, likely negated the benefits of a digital intervention (eg, its ease of access). A recently conducted RCT supports this notion [70]. The authors assessed the effectiveness of the ICare Prevent intervention on symptom reduction rather than its preventive potential. Although their inclusion and exclusion criteria were less restrictive than those reported in this study and the target sample size was eventually reached, ≤3% of all recruited students could be randomized, largely because students did not provide written informed consent. It needs to be stated that the COVID-19 pandemic has led the evaluating Medical Ethics Committee to reconsider its reluctance to allow digital informed consent. In addition, the possibility that students genuinely did not want to consent to the research procedure cannot be excluded.

In line with the aforementioned potential explanations, the individual approach through the participant platform generated by far the largest number of study sign-ups. As the platform was geared toward participation in clinical studies, students who had registered were likely already aware of health complaints, either physical or mental, both of which are often comorbid [71]. Consequently, almost all students recruited through this channel exceeded the clinical cutoff for mental health problems. It is noteworthy that the screening instrument we used (MINI) has been shown to be overly sensitive and, therefore, likely overestimated the prevalence of major depression in our sample [58]. However, the high degree of psychopathology also sheds light on another potential underlying issue, namely, students’ attitudes toward help seeking in general [31]. This problem might also explain why the most efficient approach to recruitment (ie, direct contact through email addresses) resulted in the randomization of only 35 students.

Although, in recent mental health surveys among students in the Netherlands, a response rate of <12% was found [72], detailed accounts of recruitment challenges are generally limited to treatment studies in nonstudent populations. An early systematic review of 78 studies summarized a decade of barriers to trial recruitment [73]. In line with our findings, the most common concerns were related to the information and consent procedure and the inconvenience caused by the research context (eg, strict procedures, additional appointments, and travel time, the latter being less applicable as there is no travel time and limited in-person contact with physicians or scientific staff in trials on digital interventions). Subsequent reviews have confirmed the relevance of these concerns to recruitment and participation [74,75]. In an attempt to quantify the problem, a study systematically reviewed 1017 study protocols of RCTs, most from the field of oncology and cardiovascular medicine, and compared their available recruitment and publication status [76]. Approximately one-quarter of the studies were terminated prematurely, most commonly because of recruitment challenges and, of these, only 40% were published [76]. These challenges are likely more pronounced in prevention studies [29] considering that individuals at prodromal stages of, for example, depression might not feel the urge to seek help and might prefer self-management of complaints. On the basis of these findings, a set of general recommendations [30,77] should be emphasized.

First, we encourage complementing RCTs of new interventions or in new target groups with well-designed feasibility trials [77]. A recent example is provided by a study assessing the feasibility and acceptability of the ICare Prevent intervention in Indonesian students, with positive results [78]. Importantly, such web-based interventions are novel in Indonesia, and treatment for mental health complaints is limited in the country, potentially explaining why recruitment was less problematic. Conversely, another study reported substantial challenges in recruiting participants, with the informed consent procedure again being a major barrier [79]. In this feasibility study, the ICare Prevent intervention was adapted for patients with coronary artery disease. Although such feasibility or pilot trials are regularly conducted, it is advisable to routinely extend the assessment of the feasibility, acceptability, and cost-effectiveness to the potential recruitment measures and report these in outcome papers. Therefore, a structured and preplanned evaluation of recruitment strategies is important. For example, a study indicated that recruitment via posters and websites was most cost-effective in the context of an RCT on depression relapse prevention while noting that a multifaceted recruitment strategy was crucial [80]. In our study, such a diverse set of strategies proved unsuccessful. In line with this, a review of effective approaches to recruiting participants for RCTs has shown that the quality of available evidence is low and that those effective approaches that emerged from higher-quality studies, such as unblinding trials and calling potential participants, are not applicable in many research contexts [81]. On a brighter note, the dearth of information has advanced initiatives such as the Prioritising Recruitment in Randomised Trials project [82], which entails a web-based compilation of important recruitment-related questions and answers provided by experts. It offers an open-access database of expert-based suggestions on ways to improve recruitment for clinical trials.

Second, cocreation with end users should be considered beyond intervention development. Notably, a systematic review suggested that involving participants in the creation of study information material was not efficient [81]. However, active participation of students in the identification of effective recruitment channels beyond development (eg, flyers and posters) would allow for a targeted recruitment strategy. In line with this idea, although a broad recruitment strategy was deemed most effective initially for the ICare Prevent trial, the results outlined previously show that saving efforts for specific channels is more efficient.

Finally, it has been suggested to overcome the relatively low uptake of preventive mental health interventions by focusing on indirect prevention—interventions that do not directly address mental health problems but rather target related issues (eg, insomnia) with the aim of positively affecting mental health conditions [30]. More research is needed to investigate this possibility, but examples of digital interventions that use such an indirect prevention approach exist, for example, a set of Complaint-Directed Mini-Interventions for depression, evaluated in the general population [83]. These interventions consist of short web-based modules for improving sleep, reducing stress, and tackling excessive worrying. The authors found not only a significant reduction in depressive symptoms at the 3-month follow-up compared with a waitlist control (*d*=−0.7) but also a promising sign-up rate and provision of informed consent. In the context of our study, the prominent focus on depression and anxiety might have deterred students at subclinical stages. As noted previously, these students might not have identified as having depressive or anxiety complaints, thereby reducing the need for seeking help. Moreover, in the absence of an urge to seek help, the research context (eg, informed consent, interviews, and questionnaires) might have been experienced as too burdensome. This serves as a potential explanation for why mostly those students with clinical presentations of mental health conditions signed up for the RCT.

### Intervention Use and Effects

The use of the intervention was low, with an average of 3 sessions completed by the participants. Some of the main intervention components were presented in these first sessions, such as psychoeducation and behavioral activation. However, the transdiagnostic components, which were mainly contained in the elective modules, were only provided from the second session onward, and other core elements such as cognitive restructuring followed in later sessions. Moreover, the analyses did not suggest an intervention effect on depression or anxiety outcomes. Only anecdotal evidence for the added benefit of individual guidance was obtained as the only participant who completed the booster session received support from a coach. Whether the low use of the intervention also affected the absence of intervention effects on mental health outcomes cannot be established because of the small sample size. However, these findings complement studies on the same intervention conducted in different contexts. Although one study reported positive within-group effects immediately after the intervention (depression: *d*=0.42; anxiety: *d*=1.19) [84], another study found no difference between the ICare Prevent intervention and CAU in an RCT among students [70]. Moreover, a meta-analysis on digital mental health interventions in students has suggested that the effect sizes of such interventions are small at best in this population (depression: *d*=0.18; anxiety: *d*=0.27), and subsequent trials will likely include null findings (prediction interval for depression: −0.26 to 0.62; prediction interval for anxiety: −0.36 to 0.90) [28]. Therefore, it is conceivable that the ICare Prevent intervention requires further adaptation before being suitable for students.

### Limitations and Strengths

This study has a number of limitations. The encountered recruitment challenges resulted in a small sample size, which had considerable implications. First, it meant that we could not follow the analytical approach outlined in the study protocol [40]. Additional analyses (eg, on the relationship between dropout and outcome) were not possible. Moreover, pooling the individually and automatically guided intervention conditions was a compromise to reach stable statistical models that would allow for a description of the data distribution (see also Multimedia Appendix 4). Previous research has consistently indicated the superiority of individually guided interventions, specifically those involving human rather than technical support [85], although this applies less so for subclinical stages of depression [86]. Therefore, any suggestions and findings need to be considered exploratory and incidental and require replication. However, we chose the alternative Bayesian framework to overcome the sample size limitations inherent in frequentist statistics and provide a basic account of the intervention’s effectiveness. Although we used noninformative priors, a strength of this study is that it provides a starting point for future trials. Essentially, subsequent RCTs on the topic of transdiagnostic preventive digital health interventions for students can build on our data and use them as prior information [64,87]. This would allow for a continuous research circle from feasibility trial, which generates the prior information, to full RCT, which provides up-to-date information.

Moreover, we did not use a structured evaluation of the recruitment challenges or a broader process evaluation as this was not part of the originally planned RCT. This includes a systematic appraisal of recruitment costs, which we have not reported on because of a lack of reliable data. Although this is a lesson learned for future RCTs, reporting both unforeseen recruitment challenges and data on participants and their use of the intervention is important nevertheless. For example, evidence of the effectiveness of interventions might suffer from publication bias. In this regard, a strength of this study is the publication of data that can be beneficial not only in guiding future studies on developing recruitment strategies but also in data synthesis efforts such as meta-analyses. The high costs involved in clinical trials and the intensive involvement of both staff and participants mandate such reporting of the collected data, which hopefully can aid in preventing similar situations in the future and, therefore, save scarce resources [76].

### Conclusions and Future Research

The recruitment of students for digital mental health interventions that focus on the prevention of depression and anxiety is difficult. Although targeted approaches such as direct email contact seem the most efficient, more research is needed on factors that can improve recruitment, and subsequent improved strategies need to be developed. Moreover, evidence on whether direct rather than indirect prevention efforts are suitable for this target group is mixed and requires further investigation. We provided an account of recruitment challenges as well as basic information on intervention effects that can aid future studies in the development and evaluation of similar interventions.

## Appendix

Multimedia Appendix 1 Recruitment poster.

Multimedia Appendix 2 Recruitment sticker.

Multimedia Appendix 3 Intervention description.

Multimedia Appendix 4 Sensitivity analysis on all conditions.

Multimedia Appendix 5 CONSORT-eHEALTH checklist (V 1.6.1).

## Abbreviations

BF: Bayes factor
CAU: care as usual
CI: credibility interval
CONSORT-EHEALTH: Consolidated Standards of Reporting Trials of Electronic and Mobile Health Applications and Online Telehealth
GAD: generalized anxiety disorder
GAD-7: 7-item Generalized Anxiety Disorder Scale
GP: general practitioner
MDD: major depressive disorder
MINI: Mini International Neuropsychiatric Interview
PHQ-9: 9-item Patient Health Questionnaire
QIDS-CR: Quick Inventory of Depressive Symptomatology-Clinician Rated
RCT: randomized controlled trial
SIGH-A: Structured Interview Guide for the Hamilton Anxiety Rating Scale

## Data Availability

The statistical packages in RStudio (RStudio, Inc) used were as follows: bayestestR (version 0.12.1 [88]), foreign (version 0.8-82 [89]), ggplot2 (version 3.3.5 [90]), Hmisc (version 4.7-0. [91]), and Psych (version 2.2.3. [92]). Full analytical code is available [93].
