# Supplementary figures and images for: Challenges in Recruiting University Students for Web-Based Indicated Prevention of Depression and Anxiety: Results From a Randomized Controlled Trial (ICare Prevent)
Source: J Med Internet Res. 2022 Dec 14;24(12):e40892. doi: 10.2196/40892 (PMC9798269; doi:10.2196/40892)

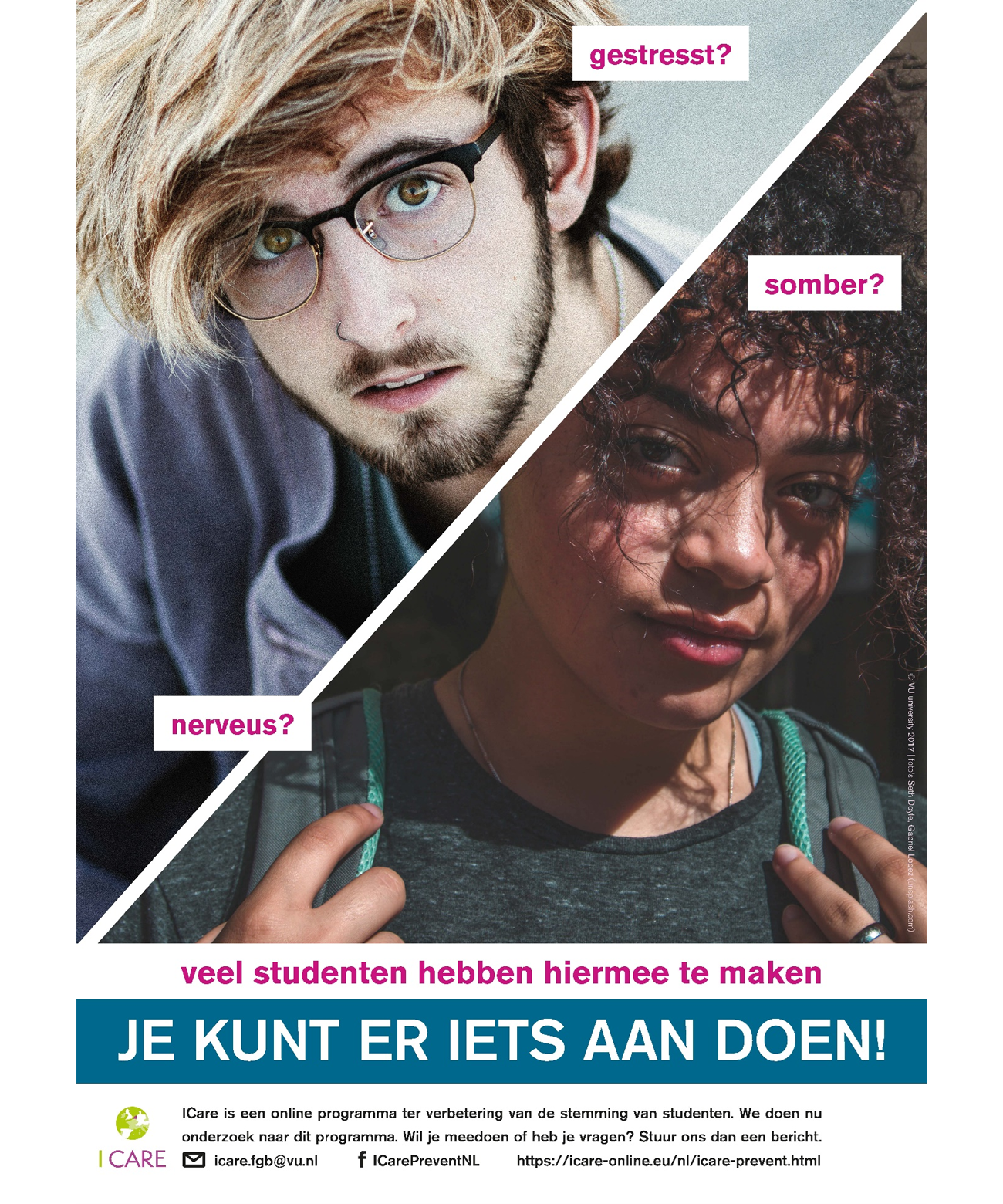

Supplement: Multimedia Appendix 1 [file jmir_v24i12e40892_app1.png]

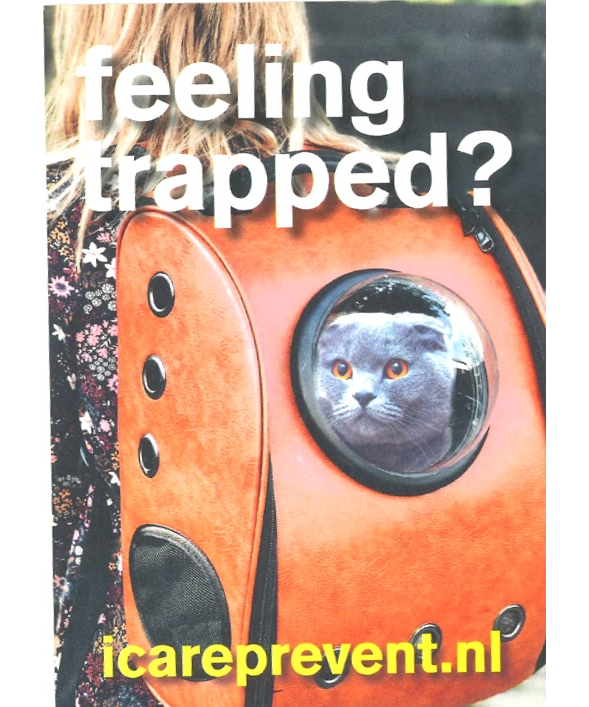

Supplement: Multimedia Appendix 2 [file jmir_v24i12e40892_app2.png]
