# Supplementary material for: Challenges in Recruiting University Students for Web-Based Indicated Prevention of Depression and Anxiety: Results From a Randomized Controlled Trial (ICare Prevent)
Source: J Med Internet Res. 2022 Dec 14;24(12):e40892. doi: 10.2196/40892 (PMC9798269; doi:10.2196/40892)
Supplement: Multimedia Appendix 3 [file jmir_v24i12e40892_app3.docx]

**Intervention description: ICare Prevent**

Session 1 introduces the (technical) functionalities of the intervention and asks users to set their goals. Next (session 2), they identify their specific problems and receive information on how to tackle these through behavioral activation. Session 3 provides psychoeducation on depression and anxiety, followed by cognitive restructuring techniques (session 4). In session 5, users prioritize their most prominent complaints and decide to follow either problem-solving (for depression) or exposure (for anxiety) techniques. These are further extended in session 6. The last core session (session 7) serves as a reflection on the things learned during the intervention and to make a plan for maintaining the use of learned techniques in the future. The booster session (session 8, accessible one month after completion of the seventh session) allows users to evaluate their maintenance strategies and to reflect on their achievements.

A number of elements were aimed at making the intervention more interactive and increasing usability. For instance, a read-aloud function was included, and additional information was generally provided through drop-down texts, giving users control over the amount of text they wanted to read. The platform allowed for linking information entered in one session to other sessions (e.g., goals entered in session 1 were displayed in subsequent sessions). Moreover, via an optional smartphone app participants could access the diaries and could opt to receive one automatic motivational message per day. Other interactive elements such as videos, audio files, and testimonials complemented the informational texts and explained homework exercises.
