# Supplementary material for: Challenges in Recruiting University Students for Web-Based Indicated Prevention of Depression and Anxiety: Results From a Randomized Controlled Trial (ICare Prevent)
Source: J Med Internet Res. 2022 Dec 14;24(12):e40892. doi: 10.2196/40892 (PMC9798269; doi:10.2196/40892)
Supplement: Multimedia Appendix 4 [file jmir_v24i12e40892_app4.docx]

**Sensitivity analysis on all conditions.**

The main analysis was repeated with intervention condition separated as individually guided vs. automatically guided ICare Prevent. The CAU condition was taken as a reference point and at each assessment point, the hypothesis was tested that the interaction effect was larger in the individually guided than in the automatically guided intervention condition, i.e.: (ICare Prevent individually guided > ICare Prevent automatically guided) > CAU. Only two models reached convergence, namely those on the clinical interview outcomes (QIDS-CR, SIGH-A). Table S1 provides an overview of parameter estimates of these two models, while the Bayes Factors for the specific hypotheses can be found in Table S2. The latter indicates that there is no evidence for the superiority of individual above automatic guidance compared to CAU. However, as with the main analysis, uncertainty is large (see also CIs in Figures S1 and S2).

Table S1. Estimates for the interaction with intervention condition (CAU= reference group) per assessment point and instrument.

| Individually guided condition | | | |
| --- | --- | --- | --- |
| Timepoint | Estimate, β | 95% CI^a^ | 95% HDI^b^ |
| QIDS-CR^c^ | | | |
|  |  |  |  |
| Baseline vs posttest assessment | -.68 | -3.25 to 1.98 | -3.39 to 1.82 |
| Baseline vs 6-month follow-up | -1.09 | -5.04 to 2.79 | -4.83 to 2.95 |
| Baseline vs 12-month follow-up | -1.70 | -4.81 to 1.45 | -4.81 to 1.44 |
| SIGH-A^d^ | | | |
|  |  |  |  |
| Baseline vs posttest assessment | 1.47 | -3.57 to 6.51 | -3.41 to 6.64 |
| Baseline vs 6-month follow-up | -4.33 | -11.24 to 2.47 | -11.07 to 2.62 |
| Baseline vs 12-month follow-up | 1.41 | -3.37 to 6.06 | -3.21 to 6.18 |
| Automatically guided condition | | | |
| QIDS-CR^c^ | | | |
|  |  |  |  |
| Baseline vs posttest assessment | -2.12 | -5.52 to 1.31 | -5.56 to 1.15 |
| Baseline vs 6-month follow-up | -3.18 | -6.84 to 0.46 | -6.92 to 0.37 |
| Baseline vs 12-month follow-up | -3.38 | -9.29 to 2.60 | -9.33 to 2.56 |
| SIGH-A^d^ | | | |
|  |  |  |  |
| Baseline vs posttest assessment | 0.97 | -5.32 to 7.31 | -5.25 to 7.35 |
| Baseline vs 6-month follow-up | -2.63 | -9.36 to 4.01 | -9.38 to 3.97 |
| Baseline vs 12-month follow-up | 2.42 | -6.73 to 11.42 | -6.63 to 11.49 |

^a^CI= Credibility Interval.

^b^HDI= High Density Interval.

^c^QIDS-CR= Quick Inventory of Depressive Symptomatology-Clinician Rated.

^d^SIGH-A= Structured Interview Guide for the Hamilton Anxiety Rating Scale.

Table S2. Bayes Factors for alternative hypothesis: (ICare Prevent individually guided > ICare Prevent automatically guided) > CAU per instrument and assessment point.

| Timepoint | *BF_10_*^a^ | *BF_01_*^b^ |
| --- | --- | --- |
| QIDS-CR^c^ | | |
| Posttest assessment | 0.21 | 4.69 |
| 6-month follow-up | 0.17 | 5.94 |
| 12-month follow-up | 0.35 | 2.88 |
| SIGH-A^d^ | | |
|  |  |  |
| Posttest assessment | 0.76 | 1.31 |
| 6-month follow-up | 2.27 | 0.44 |
| 12-month follow-up | 1.49 | 0.67 |

^a^BF_10_= Bayes Factor indicating probability of alternative over null hypothesis.

^b^BF_01_= Bayes Factor indicating probability of null over alternative hypothesis

^c^QIDS-CR= Quick Inventory of Depressive Symptomatology-Clinician Rated.

^d^SIGH-A= Structured Interview Guide for the Hamilton Anxiety Rating Scale.

**
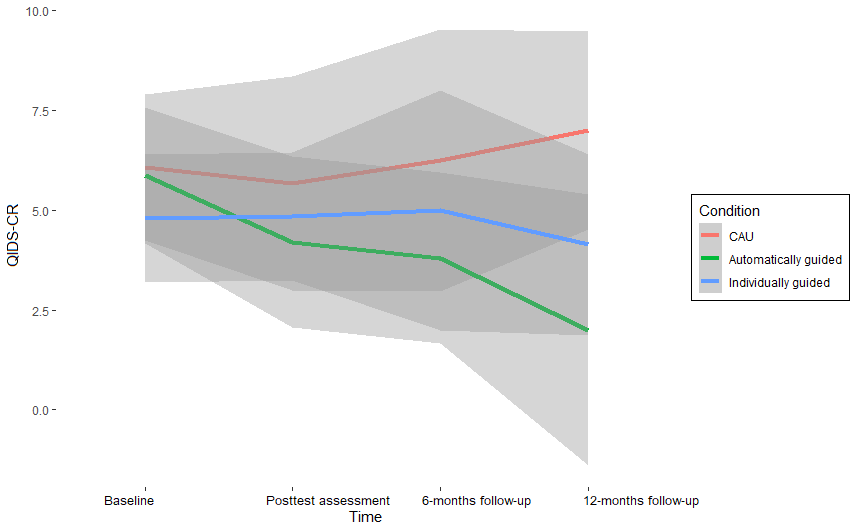
**

Figure S1 Depression scores (QIDS-CR) per assessment point and condition.

**
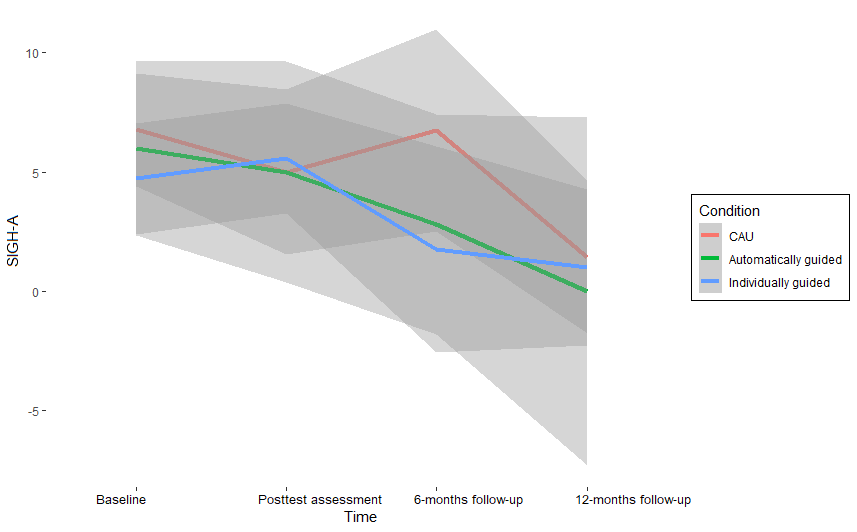
**

Figure S2 Anxiety scores (SIGH-A) per assessment point and condition.
